# SunoCaps: A novel dataset of text-prompt based AI-generated music with emotion annotations

**DOI:** 10.1016/j.dib.2024.110743

**Published:** 2024-07-18

**Authors:** M. Civit, V. Drai-Zerbib, D. Lizcano, M.J. Escalona

**Affiliations:** a Department of Communication and Education, Universidad Loyola Andalucía. *Av*. de las Universidades s/n. 41704 Sevilla, Spain; b LEAD - CNRS UMR5022 Université Bourgogne Institut Marey - I3M, 64 rue de Sully, Dijon 21000, France; c Universidad a Distancia de Madrid, Carretera de La Coruña, KM.38,500 Vía de Servicio, n° 15, Collado Villalba, Madrid 28400, Spain; d Universidad de Sevilla, ETS Ingeniería Informática, Avda. Reina Mercedes s/n, Seville 41012, Spain

**Keywords:** Data, Automatic music generation, Emotion feature, Artificial intelligence, Prompt alignment, Generative AI

## Abstract

The SunoCaps dataset aims to provide an innovative contribution to music data. Expert description of human-made musical pieces, from the widely used MusicCaps dataset, are used as prompts for generating complete songs for this dataset. This Automatic Music Generation is done with the state-of-the-art Suno generator of audio-based music. A subset of 64 pieces from MusicCaps is currently included, with a total of 256 generated entries. This total stems from generating four different variations for each human piece; two versions based on the original caption and two versions based on the original aspect description.

As an AI-generated music dataset, SunoCaps also includes expert-based information on prompt alignment, with the main differences between prompt and final generation annotated. Furthermore, annotations describing the main discrete emotions induced by the piece. This dataset can have an array of implementations, such as creating and improving music generation validation tools, training systems for multi-layered architectures and the optimization of music emotion estimation systems.

Specifications Table

**Table:** 

Subject	Computer Science/Artificial Intelligence.
Specific subject area	*The SunoCaps dataset contains text prompt-based AI generated music created with the Suno music generator. These prompts come from the MusicCaps dataset that has been used to train a wide variety of AI music generators. It also includes expert comments on the alignment of the generated music with the prompts and the emotions associated with the generated music. SunoCaps can be used for evaluating AI based Music generators, improve validation tools, and develop user-based methodologies. This is an important task in automatic music generation (AMG), as it can help to improve the quality, reliability, and user acceptance of this type of systems. SunoCaps can also be used as model training data for audio-based music generators.*
Type of data	*Raw audio Mp3 files at 192kbps and 48KHz with Lavf58.29.100 encoder* *Table (.csv file) with prompts, emotion and alignment annotations.*
Data collection	*Audio data generated with the Suno AI generator in mp3. Prompts collected from the MusicCaps dataset and minimally altered to conform to Suno specifications. Annotations collected through expert assessment.*
Data source location	*Escuela Técnica Superior de Ingeniería informática. Universidad de Sevilla. Av. Reina Mercedes s/n. 41012 Sevilla, España.*
Data accessibility	Repository name: SunoCaps Data identification number: DOI: 10.34740/kaggle/ds/4891165 Direct URL to data: http://doi.org/10.34740/kaggle/ds/4891165 Data Freely available to anyone with internet access and the web server address provided
Related research article	None

## 1 Value of the Data

• SunoCaps [1] is the first publicly available dataset where expert description of human-made musical pieces are used as prompts for Automatic Music Generation with a state-of-the-art audio-based generator. The widely used MusicCaps [2] dataset aspect and captions descriptions are used as prompts to generate four different versions with the Suno AI generator [3].
• The data includes expert annotation for prompt alignment. This offers insights into the functioning of the text-based music generators and can be used to develop validation tools that compare human and AI created music. Furthermore, the multiple versions with different annotations for each original song can be used to train automatic models for supervising music generators in multi-layered architectures.
• Emotional tagging is included in the dataset for each specific piece. These tags display the general emotion corresponding to the discrete emotion model [4] and one or more extra descriptors for a more nuanced definition. This data contributes to the research advancement in emotional assessment of AI generated music.
• Due to a general duration of around or over a minute, the dataset can be used to train and improve models for emotion recognition in music, as currently they rely on a minimum duration of the stimuli. This minimum has been established in 450 frames (i.e., 18 s at 25 fps frame rate) [5] and at 30 s for Heart Rate Variability estimations [6]. Additionally, the primary tagging is very consistent with only a few possible options which enables the optimization of models for shorter stimuli and facilitates the augmentation of the dataset in the future.
• An example iPython notebook is provided which connects the MusicCaps and SunoCaps datasets. This allows for easy development of applications that use both the SunoCaps and MusicCaps datasets.

## 2 Background

With music generation being a topic of increasing interest both for the industry and the academia [7], there is the necessity for validation tools of AI music generators and their creations. Through our research we have encountered how a music audio dataset that deal with prompt alignment problems could be very beneficial for the development of the aforementioned validation tools which propelled us to develop this dataset. The dataset from which this newly created one emanates, MusicCaps dataset, is one of the most currently used in the field of audio-based music generation. This makes the selection of said dataset a solid benchmark for testing validation tools and models with multiple generators.

Moreover, current natural-language and text-prompt based generators are very lenient on emotion descriptions of music. This fact drove us towards pursuing different modes of annotating the emotions of the generated pieces. As we decided to use human-made annotations, they are valuable both for AI training and for improving automated music emotion recognition (MER) [8]. As some MER systems require music of a minimum duration to measure significant stimuli. The minute-long generations offer a significant advantage.

## 3 Data Description

The dataset consists of a series of songs generated with SUNO, an AI music generator that outputs audio. In contrast with symbolic generators that generate a midi or similar file, similar to a score, this dataset is composed of mp3 audio files accompanied by a csv file with annotations. These songs are generated from the descriptors of the MusicCaps [2,9] dataset of human-made music.

Furthermore, the generated songs follow the name structure ``abcd\_1”. The name before the underscore corresponds to the original Youtube identifier of the MusicCaps dataset while the number after the underscores represents the version number.

Each individual version is annotated with the original prompts, indicating whether is an aspect-based or a captions-based version. Moreover, they have expert annotations on prompt alignment in every version when there are significant differences between the prompts and the generated results. This can be seen in the comment column of the csv file.

All generated versions are tagged with a main emotion derived from the discrete emotion model. On many occasions, this tagging seemed insufficient by the reviewers and a more specific emotional description is added in a separate column.

The songs curated in the dataset are from different genres, both from western and non-western music traditions and both instrumental and with lyrics depending on the version. Almost all songs are around or over a minute in duration and the rare specific exceptions to this rule are annotated in the comments.

An iPython notebook is offered in Kaggle that portrays insights into how the dataset information can be used, as well as how it can be interconnected with the MusicCaps dataset to extract relevant information from both.

The SunoCaps dataset consists of 256 generated and annotated songs derived from a subset of 64 original MusicCaps songs. This number can be increased in the future, and a circumplex-based emotion tagging added to obtain an even more nuanced description of the emotional impact of the music.

## 4 Experimental Design, Materials And Methods

**Fig. 1:**
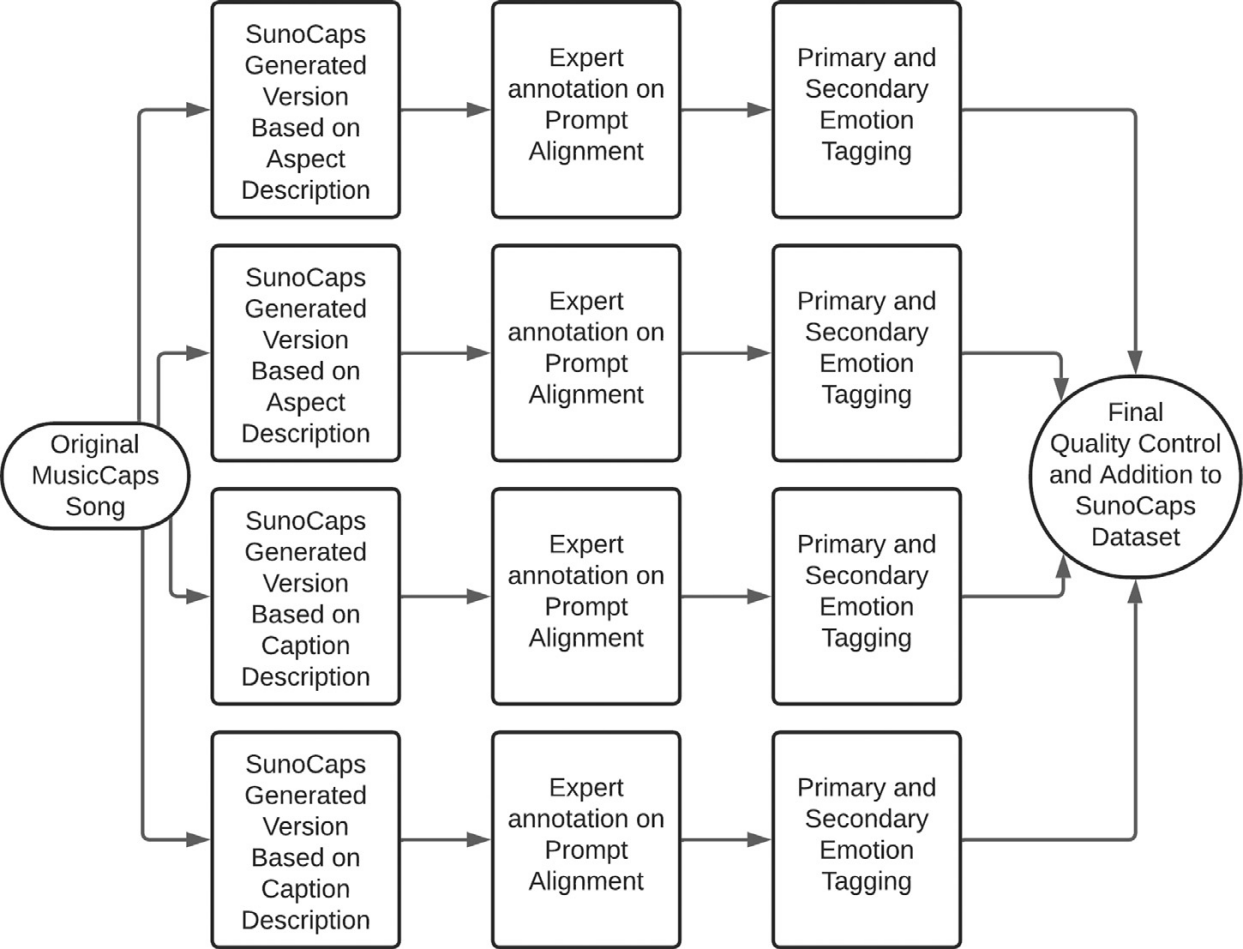
SunoCaps Song Creation Process.

The general structure of how the dataset was created can be seen in Fig. 1. The SunoCaps dataset consists of 256 generated and annotated songs derived from 64 original MusicCaps recordings.

To generate the files, we used the captions and aspect descriptions of the popular MusicCaps dataset as prompts for the Suno generator. It is worth noting that the MusicCaps dataset consists of a set of expertly annotated human-made songs from the Youtube platform. The prompts used to generate the songs are kept unchanged whenever possible and are slightly shortened, to the nearest logical sentence or caption, when reaching the maximum number of 200 characters in the Suno generator. Shortened captions or aspects are clearly marked in a separate column in the accompanying csv file.

As previously stated, the generated songs follow the name structure ``abcd\_1”. There are four songs generated from each original selected entry in the MusicCaps dataset, two based on the caption description and two generated from the aspect description. An interesting feature of this approach is the fact that the aspect description is a set of simple descriptors (like ``happy'', ``techno'', ``female voice''), while the caption description follows a natural language approach (ex. ``A female singer interprets a happy techno song''). Generations following these two different types of prompts are similar in most aspects, but they offer significant differences that can be both worth of investigation in the future and can serve as basis for comparative evaluation tools.

As a second step, the generated pieces were evaluated by human experts in music composition. This process led to the creation of the comment column in the csv of the dataset. This section deals predominantly with prompt alignment [10] problems. As such, these annotations highlight differences between the used prompts and the generated pieces and can be used to create measures of the relative space between the original prompt and the final result. These tools could also include spectral analysis or expert audition of the original audio pieces of the MusicCaps datasets from which the descriptors are derived.

As a final phase for the dataset creation, the main emotion of the pieces was tagged. This process was implemented through the agreement of three independent human raters that tagged the main emotion according to a discrete emotion model. When raters could not agree on a definitive main emotion this was resolved by adding a secondary (more nuanced and subjective) emotion tag in a secondary column named Extra_emotion. Furthermore, the implementation of a secondary emotion description, based on the continuous circumplex emotion model [11], was widely discussed among the researchers. The practical implication for model training could be interesting and a valence-arousal rating could be added in the future using Self-Assessment Mannequin-based [12].

A summarized example of the annotations and characteristics included in the csv file can be seen in Table 1. As seen in the table, for the same original song there may be different versions with good prompt alignment and therefore that have no major comments. Additionally, to help future researchers, an Instrumental column was added that provides information on whether the generated piece is instrumental or not. This is defined by the generation (or not) of lyrics, as pieces where voices are used as background instruments are usually categorized as instrumental.

**Table 1:** Sample entries from SunoCaps table.

Original Melody ytid	SunoCaps Version	Comments	Emotion	Extra_emotion	aspect version	caption version	shorth aspect	short caption	Instrumental	aspect_list	caption
−5xOcMJpTUk	−5xOcMJpTUk_1		Sad	Calm	TRUE	FALSE	TRUE	TRUE	FALSE	…	…
	−5xOcMJpTUk_2	Not clearly a male voice	Sad	Calm	TRUE	FALSE	TRUE	TRUE	FALSE	…	…
	−5xOcMJpTUk_3	Not clearly a male voice	Exciting	Energetic	FALSE	TRUE	TRUE	TRUE	FALSE	…	…
	−5xOcMJpTUk_4		Exciting	Energetic	FALSE	TRUE	TRUE	TRUE	FALSE	…	…

## Limitations

Currently the SunoCaps dataset includes 256 pieces that are generated from the caption and aspect descriptions of a subset of 64 pieces from the MusicCaps dataset. The caption and aspects of some pieces had to be shortened as the current version (V3) of the Suno generator supports only descriptions limited to 200 characters.

In the future the dataset could be expanded to include more pieces, an emotion evaluation based on the continuous circumplex model and music-theory-based annotations*.*

## Ethics Statement

The authors have read and follow the ethical requirements for publication in Data in Brief and confirm that the current work does not involve human subjects, animal experiments, or any data collected from social media platforms.

## CRediT authorship contribution statement

**M. Civit:** Investigation, Software, Validation, Writing – original draft. **V. Drai-Zerbib:** Supervision, Methodology, Writing – review & editing. **D. Lizcano:** Supervision, Funding acquisition, Writing – review & editing. **M.J. Escalona:** Conceptualization, Funding acquisition, Writing – review & editing.

## Data Availability

SunoCaps (Original data) (Kaggle).
